# Anticancer properties of 5Z-(4-fluorobenzylidene)-2-(4-hydroxyphenylamino)-thiazol-4-one

**DOI:** 10.1038/s41598-019-47177-6

**Published:** 2019-07-23

**Authors:** Konrad A. Szychowski, Danylo V. Kaminskyy, Marcin L. Leja, Anna P. Kryshchyshyn, Roman B. Lesyk, Jakub Tobiasz, Maciej Wnuk, Tadeusz Pomianek, Jan Gmiński

**Affiliations:** 1 Department of Pharmaceutical, Organic and Bioorganic Chemistry, Danylo Halytsky Lviv National Medical University, Pekarska 69, Lviv, 79010 Ukraine; 2 Department of Public Health, Dietetics and Lifestyle Disorders, Faculty of Medicine, University of Information Technology and Management in Rzeszow, Sucharskiego 2, 35–225 Rzeszow, Poland; 3 Postgraduate School of Molecular Medicine, Medical University of Warsaw, Żwirki i Wigury 61, 02–091 Warsaw, Poland; 4 Department of Genetics, Faculty of Biotechnology, University of Rzeszow, Pigonia 1, 35–310 Rzeszow, Poland; 5 Department of Management, Faculty of Administration and Social Sciences, University of Information Technology and Management, Sucharskiego 2, 35–225 Rzeszow, Poland

**Keywords:** Preclinical research, Molecular medicine, Drug development

## Abstract

4-thiazolidinones, which are privileged structures in medicinal chemistry, comprise the well-known class of heterocycles and are a source of new drug-like compounds. Undoubtedly, the 5-bulky-substituted-2,4-thiazolidinediones - a class of antihyperglycemic glitazones, which are peroxisome proliferator-activated receptor gamma (PPARγ) agonists, are the most described group among them. As there are various chemically distinct 4-thiazolidinones, different subtypes have been selected for studies; however, their main pharmacological profiles are similar. The aim of this study was to evaluate the anticancer activity of 5*Z*-(4-fluorobenzylidene)-2-(4-hydroxyphenylamino)-thiazol-4-one (Les-236) in four human cancer cell lines, A549, SCC-15, SH-SY5Y, and CACO-2, and investigate its impact on the production of reactive oxygen species (ROS) and the apoptotic process as well as cytotoxicity and metabolism in these cell lines. The cell lines were exposed to increasing concentrations (1 nM to 100 µM) of the studied compound for 6, 24, and 48 h, and later, ROS production, cell viability, caspase-3 activity, and cell metabolism were examined. The obtained results showed that the studied compound decreased the production of ROS, increased the release of lactate dehydrogenase, and decreased cell metabolism/proliferation in all the five cell lines at micromolar concentrations. Interestingly, over a wide range of concentrations (from 1 nM to 100 µM), Les-236 was able to increase the activity of caspase-3 in BJ (after 6 h of exposure), A549, CACO-2, and SCC-15 (after 48 h of exposure) cell lines which could be an effect of the activation of PPARγ-dependent pathways.

## Introduction

**Figure 1:**
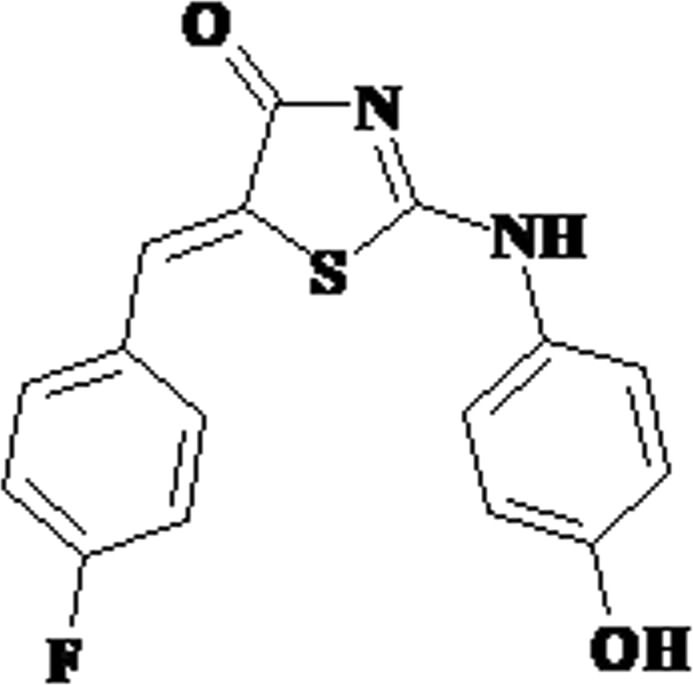
Structure of the studied compound Les-236.

4-thiazolidinones are a widely studied class of heterocycles, which are considered as privileged structures in medicinal chemistry, and a source of new drug-like compounds^1,2^_._ Undoubtedly, the 5-bulky-substituted-2,4-thiazolidinediones, a class of antihyperglycemic glitazones, which are peroxisome proliferator-activated receptor gamma (PPARγ) agonists, are the most described group among them^3^. As there are various chemically distinct 4-thiazolidinones, different subtypes have been selected for investigations; however, their main pharmacological profiles are similar and they are known to exhibit anticancer, anti-inflammatory, antiviral, and antimicrobial activity. Moreover, different 4-thiazolidinones (2,4-thiazolidinone, rhodanine, 2-amino(imino)-4-thiazolidinone, etc.), and their related (hydantoin) cores are considered as bioisosteres. 5-ene-2-amino(imino)-4-thiazolidinones represent one of the most studied subtypes of 4-thiazolidinones. The importance of C5-ene fragment had been proved in many studies^4–6^. Moreover, conjugation of the C5 exocyclic double bond to the C4 carbonyl group makes these compounds electrophilic and potentially reactive in Michael addition. This is one of the reasons why 5-ene-4-thiazolidinones, especially rhodanines, are called as pan-assay interference compounds^7–9^. This concept is still under debate; however, many useful properties of Michael acceptors have been proposed^7,9,10^: Michael acceptors, among the most effective activators of Nrf2, open new perspectives in the treatment of inflammation and cancer^11^. They act as inducers of phase 2 enzymes and a group of inducible proteins^12^ and covalent inhibitors^13^. Moreover, Michael acceptors function as reactive electrophilic species, one of the key signaling species^14^. Among the 5-ene-2-amino(imino)-4-thiazolidinones, active anticancer agents which are capable of inhibiting the growth of cancer cells were identified both in screening campaigns and small-scale studies^15–21^. Many ligands exhibiting high affinity to the known anticancer targets were also described, including integrin αVβ3 antagonists^22^, inhibitors of CDK1/cyclin B^23,24^, PPAR antagonists^25^, estrogen-related receptor-α modulators^26^, SHP-2 inhibitors^27^, PTP1B inhibitors^28^, DNA-binding agents (which bind with the DNA minor groove involving van der Waals, H-bonding, and hydrophobic interactions)^29^, and so on. Very few attempts have been made to outline the main molecular modes of action of 5-ene-2-amino(imino)-4-thiazolidinones^1,30^. Analyses of the molecular mechanisms of their action revealed their role in the following processes: induction of apoptosis, cell cycle arrest^31–33^, PPAR-related pathways^20,25^, inhibition of necroptosis^34,35^, and modulation of Red/Ox signaling and production of reactive oxygen species (ROS). Sometimes contrasting results are presented for such compounds; for example, one study reported that these compounds decrease the level of ROS^36^, whereas some other studies reported that the action of these compounds is associated with increase in the production of ROS^32,37^. The PPAR-related mechanism behind the anticancer effect was actively discussed following the identification of the role of ligand-activated transcription factor. PPARγ regulates diverse functions, such as the development of fat cells and their capacity to store lipids, expression of genes related to metabolic processes, inflammation, cell migration, apoptosis, and cell sensitivity to insulin. Moreover, in 1997, the first published reports highlighted PPARγ as a novel cancer therapeutic target regulating the differentiation of cancer cells^38,39^. Glitazones induce a variety of favorable changes in several malignancies, including liposarcoma and cancers of various organs such as breast, colon, pancreas, and prostate^40,41^. *In vitro* exposure of tumor cells to high doses of troglitazone and ciglitazone was found to lead to cell cycle arrest, apoptosis, and redifferentiation^42–44^, suggesting the involvement of PPARγ signaling pathways. Furthermore, the *in vivo* anticancer efficacy of troglitazone was demonstrated in a few clinical studies that involved patients with liposarcomas or prostate cancer^45^. The precise mechanism of antiproliferative activities of PPARγ agonists remains unclear^43^; increasing evidence indicates that 4-thiazolidinones mediate PPARγ-independent antitumor effects by targeting a wide range of signaling pathways controlling the proliferation and survival of cancer cells^46^. Analyses of the effects of 5-ene-2-amino(imino)-4-thiazolidinones also revealed the importance of these metabolic pathways in anticancer activity^1,2^. Additionally, it should be highlighted that anticancer agents such as 5-[(4-methylphenyl)methylene]-2-(phenylamino)-4(5 H)-thiazolone (MMPT) and 5-(2,4-dihydroxybenzylidene)-2-(phenylimino)-1,3-thiazolidine are also effective in the treatment of multidrug-resistant cancer^18,31,47^. The structure of MMPT has been considered as a “pharmacophore model” for further optimization^21^, and a set of structural requirements were proposed: an unsubstituted N3 atom in the main core, a C5 *p*-substituted-arylidene moiety, and a C2-arylamino fragment which can be substituted in different positions^18^. Moreover, MMPT analogs were found to exert anti-inflammatory activity through COX/LOX inhibition^48^. Other different activities of the related compounds are also being studied. The pharmacological profiles of compounds with anticancer effect and compounds exerting a combination of anticancer, antioxidation, anti-inflammatory^1,49^, and antiprotozoal^50^ effects are gaining interest. The present work is an extension of our ongoing efforts in the search for new 4-thiazolidinone-based anticancer agents^20,37,51^. Therefore, the aim of this study was to evaluate the anticancer activity of 5*Z*-(4-fluorobenzylidene)-2-(4-hydroxyphenylamino)-thiazol-4-one (Les-236) in four human cancer cell lines A549, SCC-15, SH-SY5Y, and CACO-2, and investigate its impact on ROS production and the apoptotic process as well as cytotoxicity and metabolism in these cell lines (Fig. 1).

## Results

### Les-236 decreases ROS production in cancerous cell lines

**Figure 2:**
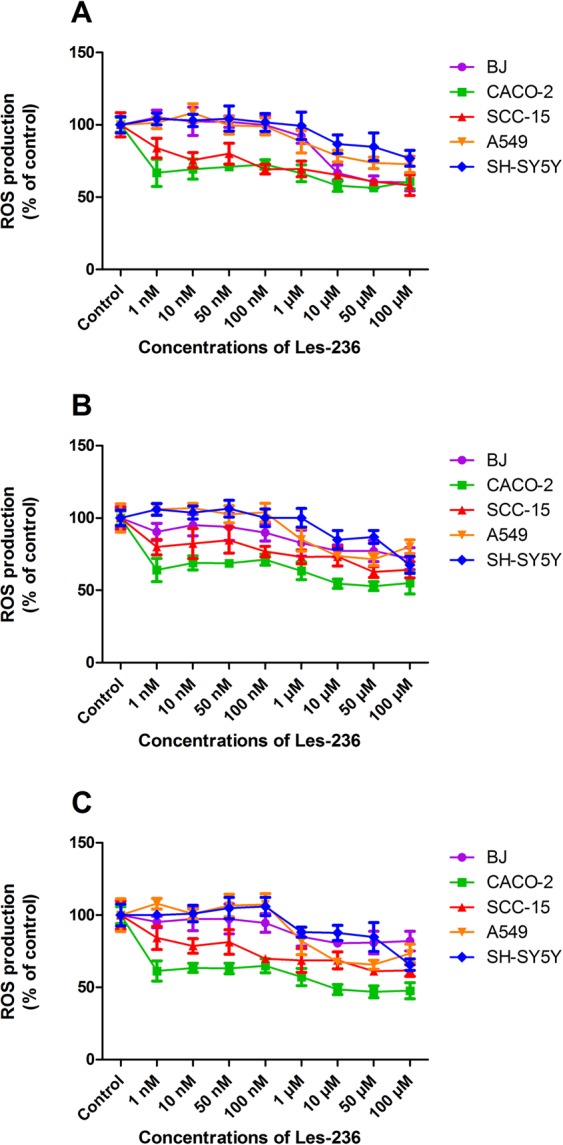
Effect of Les-236 on ROS production in cultured human cell lines after (**A**) 3, (**B**) 6, and (**C**) 24 h of exposure. The data are presented as means ± SD of three independent experiments. Each treatment was repeated six times (n = 6), and the values were measured in triplicate.

After 3 h of exposure, only 10, 50, and 100 µM concentrations of Les-236 were found to decrease ROS production in the BJ, A549, and SH-SY5Y cell lines by 14.37–39.37% compared to control. In CACO-2 and SCC-15 cell lines, Les-236 decreased ROS production at all the studied concentrations (1 nM to 100 µM) by 17.10–42.83% compared to control (Fig. 2A). Similarly, in the SH-SY5Y cell line, after 6 h of exposure only 10, 50, and 100 µM concentrations of Les-236 decreased ROS production by 16.05–33.29% compared to control. In BJ and A549 cells, Les-236 decreased ROS production at all micromolar concentrations tested (1, 10, 50, and 100 µM) by 18.33–29.13% compared to control. In CACO-2 and SCC-15 cell lines, the effect of Les-236 was more pronounced and a decrease in ROS production by 21.12–46.95% was observed compared to control (Fig. 2B). After 24 h incubation with BJ, A549, and SH-SY5Y cell lines, Les-236 decreased ROS production by 18.93–35.29% at all micromolar concentrations tested (1, 10, 50, and 100 µM) compared to control. Similarly, Les-236 decreased ROS production at all the studied concentrations in CACO-2 and SCC-15 cell lines (by 16.82–53.35%, compared to control) (Fig. 2C).

**Figure 3:**
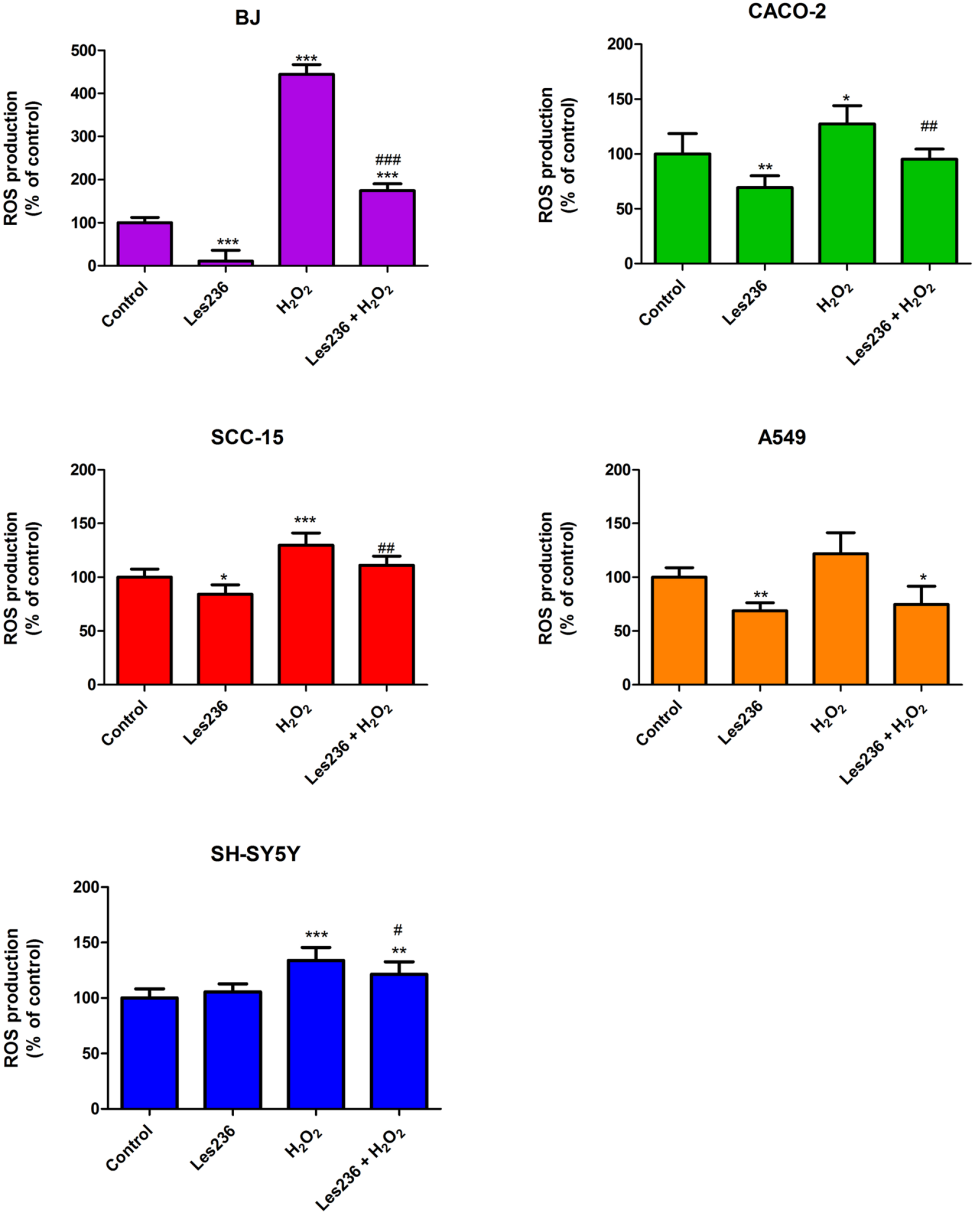
Effect of Les-236 and H_2_O_2_ on ROS production in cultured human cell lines after 6 h of exposure. The data are presented as means ± SD of three independent experiments. Each treatment was repeated six times (n = 6), and the values were measured in triplicate. *p < 0.05, **p < 0.01, ***p < 0.001, versus the control cells; ^#^p < 0.05, ^##^p < 0.01, ^###^p < 0.001, H_2_O_2_ treated cells versus the H_2_O_2_ and Les-236 treated cells.

The conducted experiments showed that after 6 h of cell co-treatment with Les-236 and hydrogen peroxide (H_2_O_2_), ROS production stimulated by H_2_O_2_ was significantly decreased by Les-236. The strongest effect of Les-236 was observed in BJ cells and the weakest in A549 cells (Fig. 3).

### Les-236 increases lactate dehydrogenase (LDH) release from cancerous cell lines

**Figure 4:**
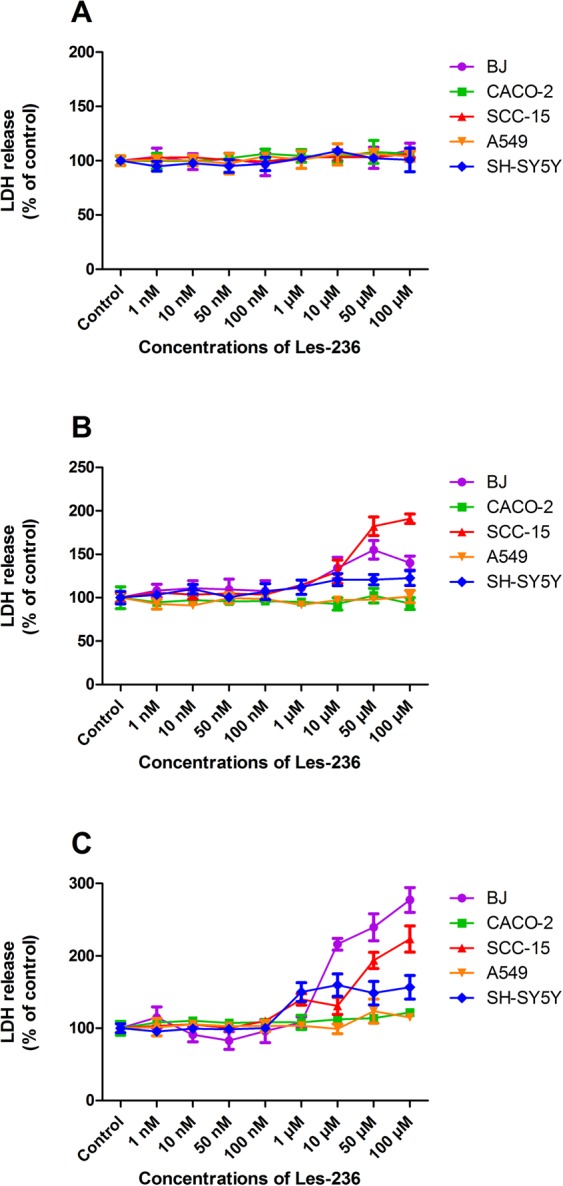
Effect of Les-236 on the release of lactate dehydrogenase (LDH) in cultured human cell lines after (**A**) 3, (**B**) 6, and (**C**) 24 h of exposure. The data are presented as means ± SD of three independent experiments. Each treatment was repeated six times (n = 6), and the values were measured in triplicate.

After 6 h of exposure to Les-236, LDH release was not observed in any of the studied cell lines (Fig. 4A). However, after 24 h of exposure, we observed an increase in LDH release at 10, 50, and 100 µM concentrations of Les-36 in SCC-15, BJ, and SH-SY5Y cell lines. In the SCC-15 cell line, LDH release was increased by 29.85%, 82.28%, and 90.92%, respectively, compared to control. In the BJ cell line, LDH release was increased by 34.28%, 55.10%, and 40.00%, respectively, compared to control. In the SH-SY5Y cell line, LDH release was increased by 20.81%, 20.81%, and 22.54%, respectively, compared to control (Fig. 4B). After 48 h of exposure to Les-236, we observed an increase in LDH release at concentrations of 1, 10, 50, and 100 µM in SCC-15 and SH-SY5Y cell lines. In the SCC-15 cell line, LDH release was increased by 39.93%, 30.82%, 93.90%, and 123.40%, respectively, compared to control. In the SH-SY5Y cell line, LDH release was increased by 50.00%, 59.70%, 48.51%, and 56.72%, respectively, compared to control. In the BJ and CACO-2 cell lines, Les-236 stimulated LDH release occurred only at the highest micromolar concentrations (10, 50, and 100 µM). In the BJ cell line, LDH release was increased by 116.10%, 239.51%, and 177.32%, respectively, compared to control. In the CACO-2 cell line, LDH release was increased by 12.16%, 13.85%, and 21.96%, respectively, compared to control. In the A549 cell line, only at 50 and 100 µM concentration of Les-36, an increase (Fig. 4C) in LDH release was observed (23.67% and 14.89%, respectively, compared to control). The half maximal effective concentrations (EC_50_) were calculated for LDH release to determine the dose-dependent effect on cell lethality (Table 1).

**Table 1:** IC_50_ and EC_50_ value (in µM of Les-236) for resazurin reduction assay, caspase-3 activity assay and LDH release assay after 48 h.

assay	IC_50_ (µM)				
	BJ	CACO-2	SCC-15	A549	SH-SY5Y
**resazurine**	48.34^c^ (±2.07)	12.71^b^ (±1.06)	64.23^d^ (±5.10)	2062.00^e^ (±16.02)	0.24^a^ (±0.02)
**assay**	EC_50_ (µM)				
**LDH**	5.59^b^ (±0.04)	nd	nd	nd	0.37^a^ (±0.04)
**caspase-3**	29.52^b^ (±2.14)	10.36^a^ (±1.54)	nd	nd	12.93^a^ (±1.82)

Different lower case letters for each assay indicate significant differences (p < 0.05). nd – not detected.

### Les-236 decreases resazurin reduction in cancerous cell lines

**Figure 5:**
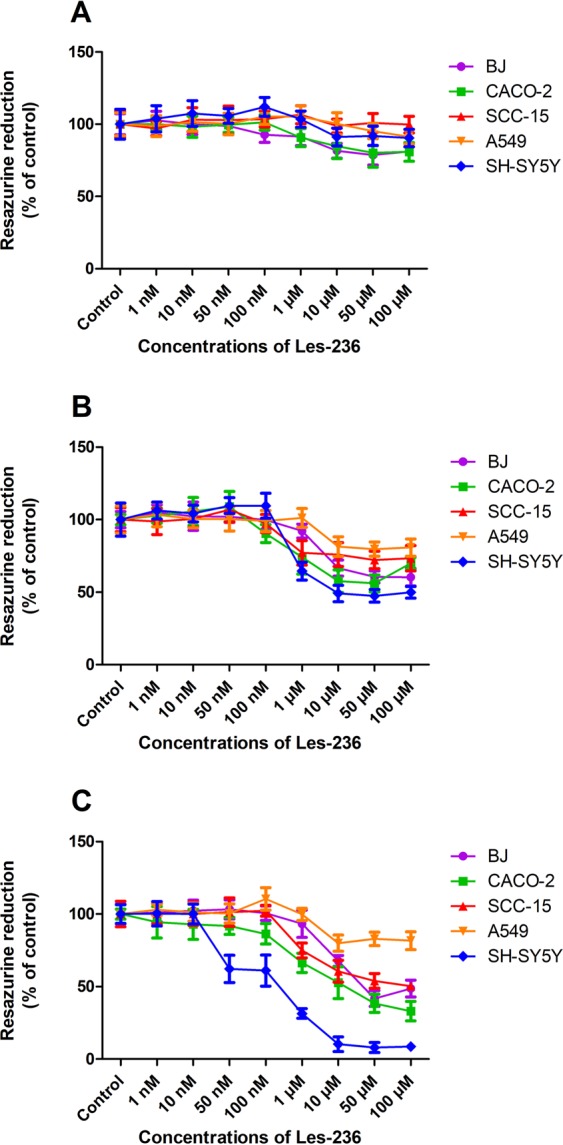
Effect of Les-236 on resazurin reduction in cultured human cell lines after (**A**) 3, (**B**) 6, and (**C**) 24 h of exposure. The data are presented as means ± SD of three independent experiments. Each treatment was repeated six times (n = 6), and the values were measured in triplicate.

After 6 h of exposure to Les-236, we observed a decrease in resazurin reduction by 18.25–19.08% in BJ and CACO-2 cell lines compared to control at concentrations of 10, 50, and 100 µM. In SH-SY5Y, A549, and SCC-15 cell lines, we did not observe any change in resazurin reduction (Fig. 5A). After 24 h of exposure to Les-236, we observed a decrease in resazurin reduction in A549 and BJ cell lines at 10, 50, and 100 µM (by 19.59%, 20.24%, and 19.23%, respectively, for A549 and by 33.35%, 39.44%, and 39.66%, respectively, for the BJ cell line, compared to control). In other cell lines (CACO-2, SCC-15, and SH-SY5Y), Les-236 decreased resazurin reduction at all the studied micromolar concentrations (1, 10, 50, and 100 µM) by 22.83–50.04% (Fig. 5B). After 48 h of exposure to Les-236, a decrease in resazurin reduction was observed in BJ and A549 cell lines at 10, 50, and 100 µM concentrations (by 20.01–51.36% compared to control). In the SCC-15 cells, Les-236 decreased resazurin reduction at 1, 10, 50, and 100 µM concentrations by 25.06%, 39.44%, 46.02%, and 49.71% compared to control. In the CACO-2 cell line, Les-236 decreased resazurin reduction at concentrations from 100 nM to 100 µM by 13.66–66.92%, compared to control. The strongest decrease in resazurin reduction was observed in the SH-SY5Y cell line. Les-236 decreased the reduction of resazurin by 37.80–91.32% at concentrations from 50 nM to 100 µM compared to control (Fig. 5C). The strongest potential of Les-236 to inhibit cell metabolism/proliferation measured by resazurine reduction was noted in the SH-SY5Y cell line at 0.24 µM and the weakest in the A549 cell line at 2062.00 µM (Table 1).

### Les-236 increases caspase-3 activity in cancerous cell lines

**Figure 6:**
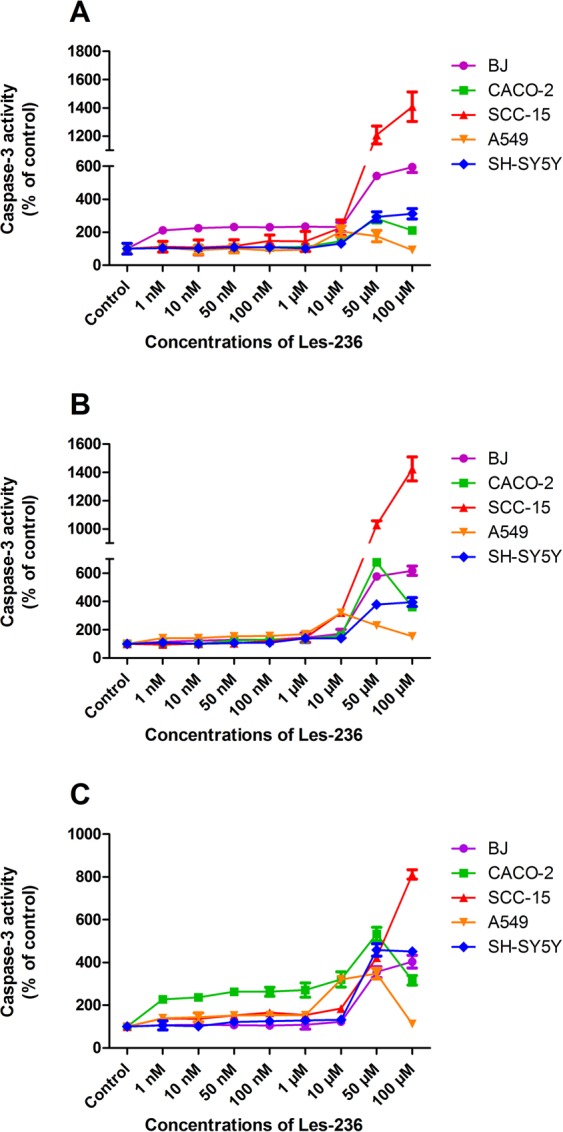
Effect of Les-236 on caspase-3 activity in cultured human cell lines after (**A**) 3, (**B**) 6, and (**C**) 24 h of exposure. The data are presented as means ± SD of three independent experiments. Each treatment was repeated six times (n = 6), and the values were measured in triplicate.

After 6 h of exposure of CACO-2 and SH-SY5Y cell lines to Les-236, we observed an increase in caspase-3 activity by 182.20–212.10% compared to control at concentrations of 50 and 100 µM. Interestingly, in the A549 cell line, an increase in caspase-3 activity was observed at concentrations of 10 and 50 µM (by 104.90% and 76.54%, respectively, compared to control). In the SCC-15 cell line, Les-236 increased caspase-3 activity at 10, 50, and 100 µM concentrations (by 128.05%, 1108.15%, and 1308.54%, respectively, compared to control). The strongest increase in caspase-3 activity by Les-236 was observed in the BJ cell line where Les-236 increased caspase-3 activity at all studied concentrations (1 nM to 100 µM) by 111.42–495.56% compared to control (Fig. 6A). After 24 h of exposure of the SH-SY5Y cell line to Les-236, an increase in caspase-3 activity was observed at concentrations of 50 and 100 µM (by 278.22% and 295.34%, respectively, compared to control). In the CACO-2 cell line, Les-236 increased caspase-3 activity at 10, 50, and 100 µM concentrations by 60.00%, 578.34%, and 261.74%, respectively, compared to control. In BJ and SCC-15 cell lines, Les-236 increased the caspase-3 activity at all the studied micromolar concentrations (1, 10, 50, and 100 µM) by 42.42–1324.24%, compared to control. Interestingly, Les-236 increased caspase-3 activity in the A549 cell line at all the studied concentrations by 40.00–220.00% compared to control (Fig. 6B). After 48 h of exposure of the BJ cell line, Les-236 increased the activity of caspase-3 (by 255.11% and 304.04%, respectively, compared to control) only at the highest micromolar concentrations of 50 and 100 µM. In the SH-SY5Y cell line, Les-236 increased caspase-3 activity at concentrations of 10, 50, and 100 µM (31.76%, 358.82%, and 351.05%, respectively, compared to control). In A549 cell line, Les-236 increased caspase-3 activity at concentrations from 1 nM to 50 µM (by 39.26–248.15%, compared to control). In CACO-2 and SCC-15 cell lines, Les-236 increased caspase-3 activity at all studied concentrations (1 nM to 100 µM) by 39.13–711.64%, compared to control (Fig. 6C). EC_50_ was calculated for caspase-3 activity to determine the dose-dependent effect on cell lethality (Table 1).

### Les-236 affects mRNA expression of *PPARγ* in cancerous cell lines

**Figure 7:**
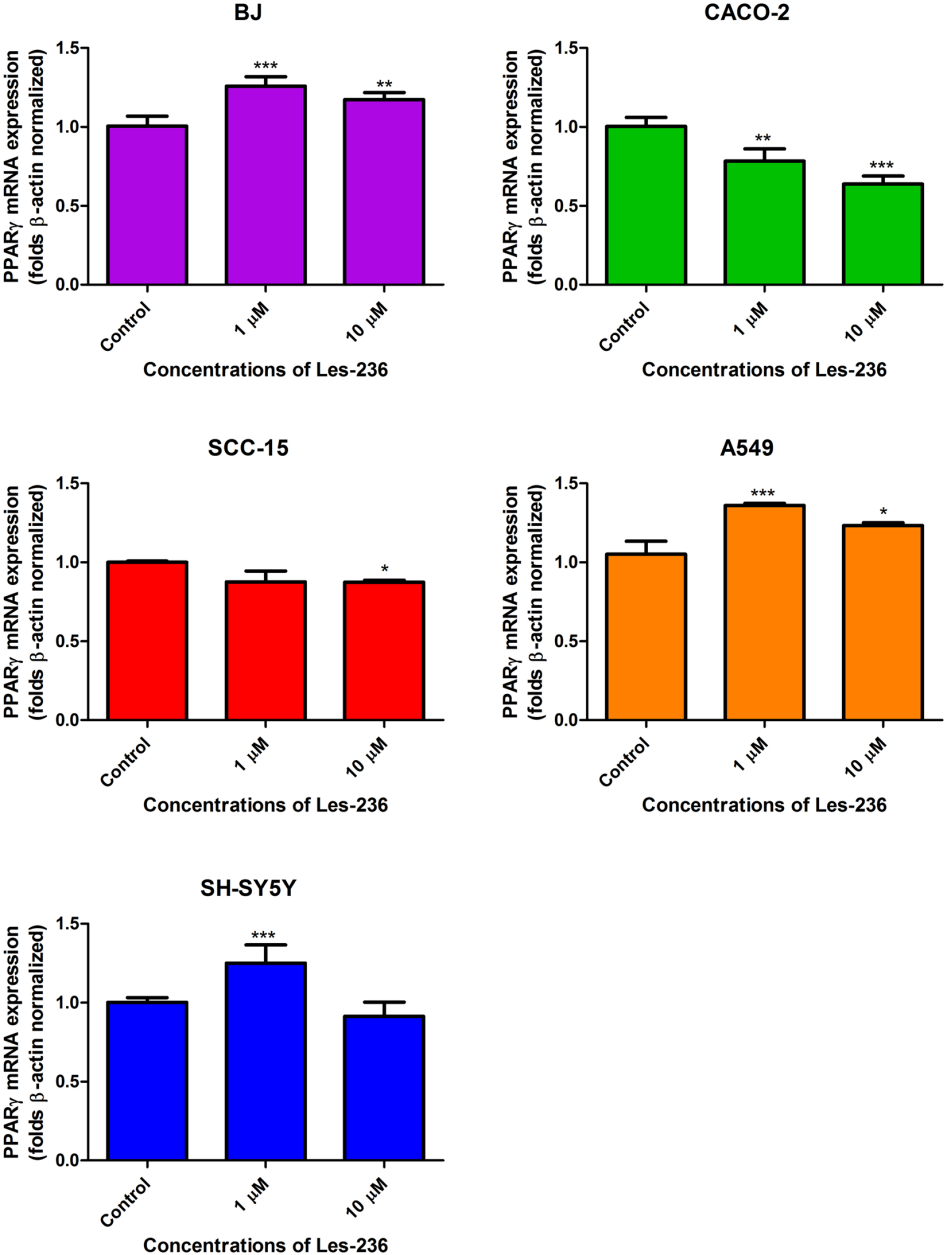
Effect of 1 and 10 μM Les-236 on mRNA expression of *PPARγ* after 6 h of exposure in cultured human cell lines after mRNA expression was normalized to *β-actin*. The data are expressed as means ± SD of three independent experiments, each of which consisted of six replicates per treatment group. *p < 0.05; **p < 0.01; ***p < 0.001 vs. the vehicle control.

After 6 h of exposure of the BJ cell line to 1 and 10 µM concentrations of Les-236, there was an increase in the expression of PPARγ by 25.76% and 17.31%, respectively. In the A549 cell line, 1 and 10 µM concentrations of Les-236 similarly increased expression of PPARγ by 36.03% and 23.33%, respectively. In the SH-SY5Y cell line, an increase in PPARγ expression was observed after treatment with 1 µM Les-236 (increased by 25.03% compared to control). In the SCC-15 cell line, a decrease in PPARγ expression was observed after treatment with 1 µM Les-236 (decreased by 12.26% compared to control). In the CACO-2 cell line, 1 and 10 µM concentrations of Les-236 decreased the expression of PPARγ by 21.67% and 36.25%, respectively (Fig. 7).

### Les-236 affects protein expression of PPARγ and KI67 in cancerous cell lines

**Figure 8:**
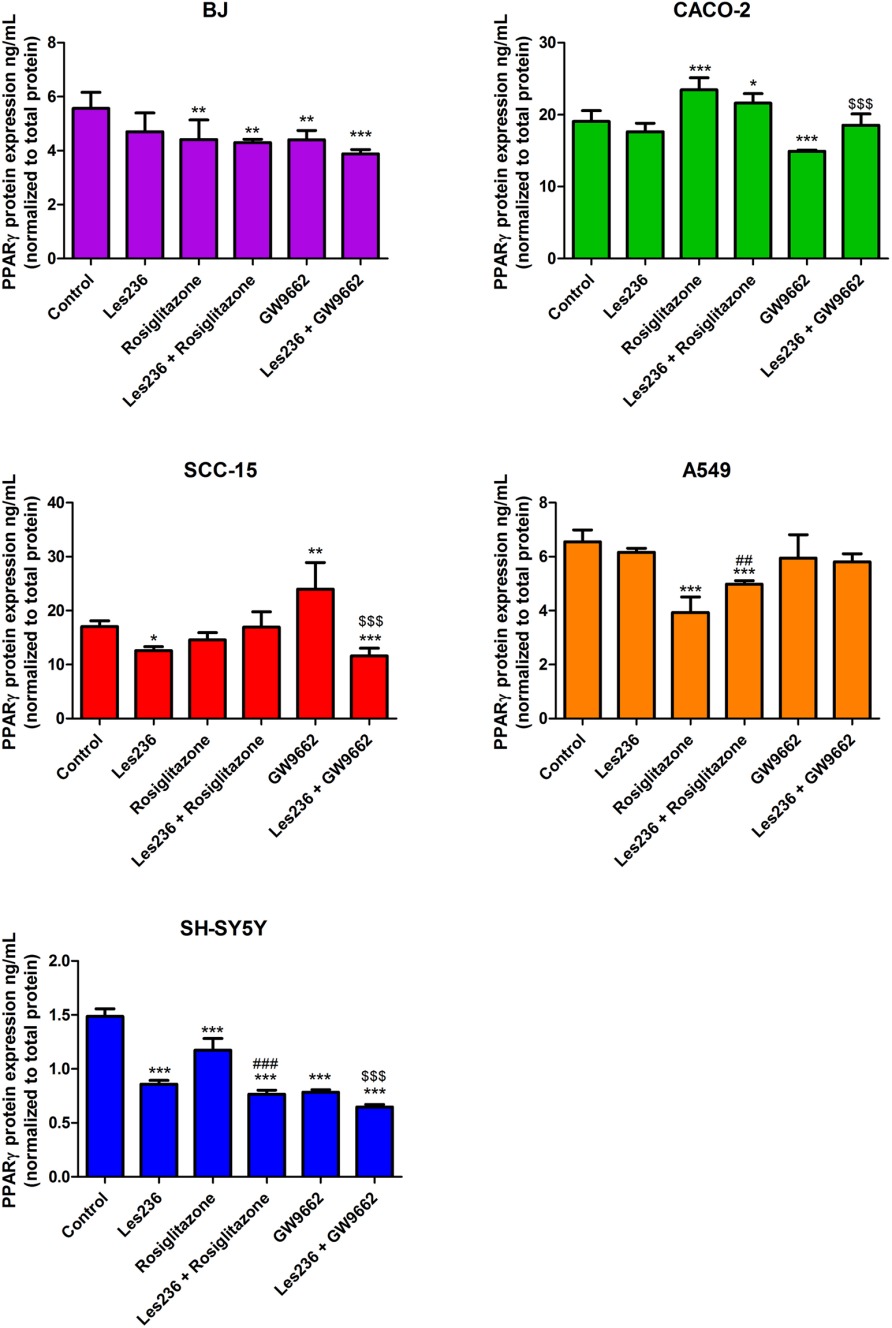
Effect of 1 µM Les-236 or co-treatment with 10 µM rosiglitazone or 10 µM GW9662 on protein expression of PPARγ after 24 h of exposure. Protein expression was normalized to the total protein level. Data are expressed as means ± SD of three independent experiments. *p < 0.05; **p < 0.01; ***p < 0.001 vs. vehicle control. ^##^p < 0.01; ^###^p < 0.001 rosiglitazone vs. Les236 with rosiglitazone. ^$$$^p < 0.001 GW9662 vs. GW9662 treated cells.

After 48 h exposure of the BJ cell line to 1 µM Les-236, no changes in the PPARγ protein expression were observed. Both the agonist (rosiglitazone) and the antagonist (GW9662) of PPARγ decreased protein expression of PPARγ (1.15 and 1.16 ng/mL, respectively, compared to control). Cell co-treatment with Les-236 and rosiglitazone or Les-236 and GW9662 did not affect expression of PPARγ compared to rosiglitazone alone or GW9662 alone, respectively. Similarly, in CACO-2 cells, 1 µM Les-236 did not affect expression of PPARγ. Rosiglitazone increased PPARγ protein expression by 5.83 ng/mL. GW9662 decreased PPARγ protein expression in CACO-2 cells by 2.03 ng/mL. Les-236 prevented decrease in PPARγ protein expression caused by GW9662. In SCC-15 cells, 1 µM Les-236 decreased in PPARγ expression by 4.47 ng/mL. Interestingly, GW9662 increased PPARγ expression by 6.96 ng/mL. Les-236 decreased expression of PPARγ stimulated by GW9662 (decreased by 12.42 ng/mL compared to GW9662 stimulated cells). After the exposure of A549 cells to 1 µM Les-236, no changes in the PPARγ protein expression were observed. Cell co-treatment with Les-236 and rosiglitazone increased PPARγ protein expression compared to the rosiglitazone treated group (increased by 1.04 ng/mL). After 48 h of exposure of the SH-SY5Y cell line to 1 µM Les-236, a decrease in the PPARγ protein expression was observed (decreased by 0.63 ng/mL). Cell stimulation with rosiglitazone and GW9662 decreased PPARγ expression by 0.31 and 0.70 ng/mL, respectively compared to control. Cell co-stimulation with Les-236 and rosiglitazone or Les-236 and GW9662 enhanced the effect of rosiglitazone or GW9662 alone (Fig. 8).

**Figure 9:**
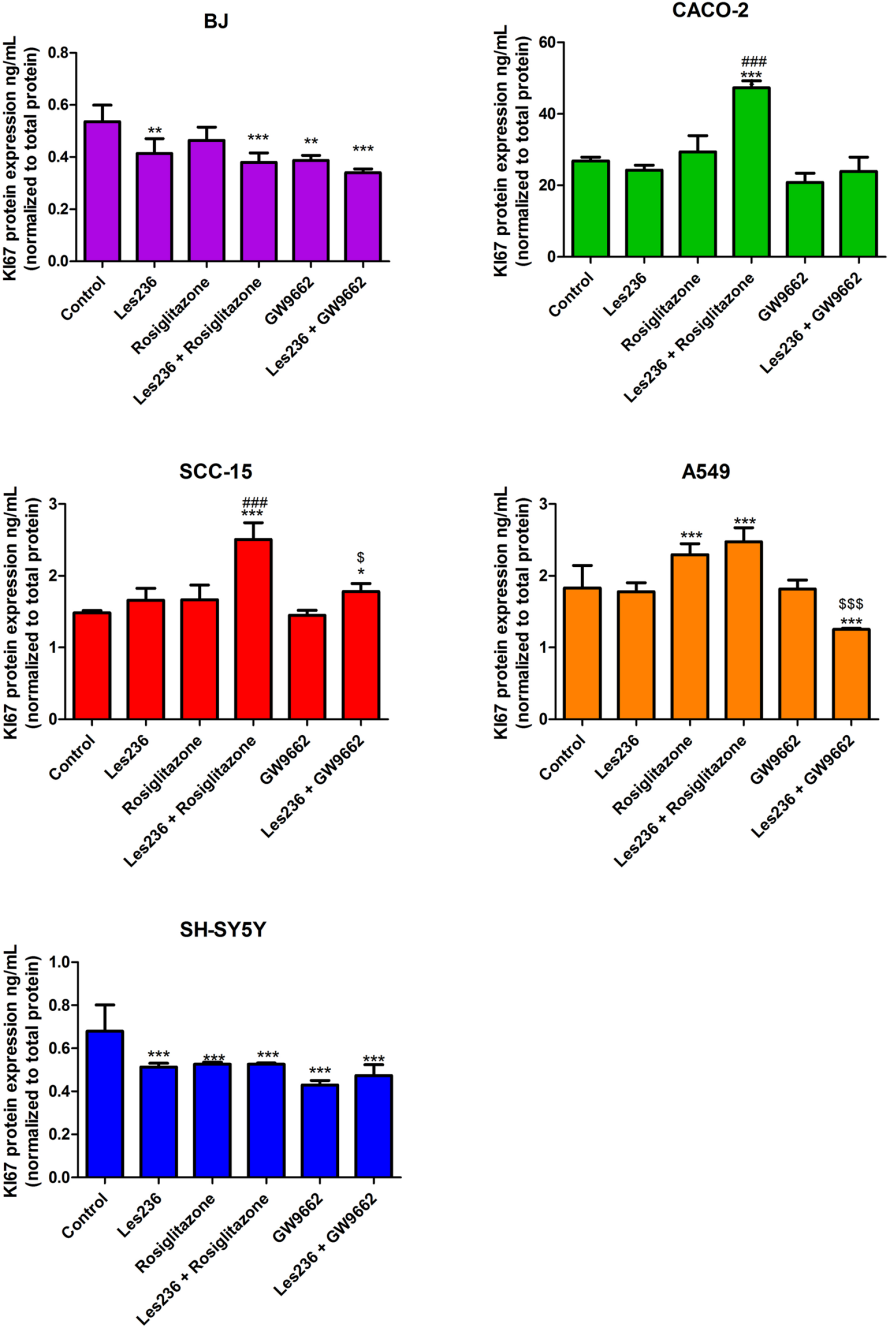
Effect of 1 µM Les-236 or co-treatment with 10 µM rosiglitazone or 10 µM GW9662 on protein expression of KI67 after 24 h of exposure. Protein expression was normalized to the total protein level. Data are expressed as means ± SD of three independent experiments. *p < 0.05; **p < 0.01; ***p < 0.001 vs. vehicle control. ^###^p < 0.001 rosiglitazone vs. Les236 with rosiglitazone. ^$^p < 0.05; ^$$$^p < 0.001 GW9662 vs. GW9662 treated cells.

After 48 h exposure of the BJ cell line to 1 µM Les-236, KI67 protein expression decreased by 0.12 ng/mL, compared to control. Rosiglitazone did not affect KI67 protein expression. GW9662 decreased protein expression of KI67 by 0.15 ng/mL compared to control. Cell co-treatment with Les-236 and rosiglitazone or Les-236 and GW9662 did not affect expression of KI67 compared to rosiglitazone alone or GW9662 alone, respectively. In CACO-2 cells, 1 µM Les-236 did not affect expression of KI67. Rosiglitazone increased PPARγ protein expression by 9.42 ng/mL. Les-236 potentiated the effect of rosiglitazone by 11.01 ng/mL compared to the rosiglitazone treated group. GW9662 decreased KI67 protein expression in CACO-2 cells by 6.01 ng/mL. Les-236 prevented decrease in KI67 protein expression caused by GW9662. In SCC-15 cells, Les-236, rosiglitazone or GW9662 alone did not affect KI67 protein expression. Interestingly, SCC-15 cell co-treatment with rosiglitazone and Les-236 or GW9662 and Les-236 increased in KI67 expression by 1.02 and 0.29 ng/mL, respectively compared to control. After A549 cell line exposure to 1 µM Les-236, no changes in the KI67 protein expression were observed. Rosiglitazone alone increased in KI67 protein expression (increased by 0.47 ng/mL compared to control); however cell co-treatment with Les-236 and rosiglitazone did not affect KI67 expression, compared to the rosiglitazone treated group. GW9662 did not affect KI67 protein expression in A549 cells but cell co-treatment with Les-236 and GW9662 decreased expression of KI67 (decreased by 0.57 ng/mL, compared to GW9662 treated group). In the SH-SY5Y cell line, 1 µM Les-236 decreased KI67 protein expression by 0.16 ng/mL, compared to control. Cell stimulation with rosiglitazone and GW9662 decreased PPARγ expression by 0.15 and 0.25 ng/mL, respectively compared to control. Cell co-stimulation with Les-236 and rosiglitazone or Les-236 and GW9662 did not affect the expression of KI67 compared to rosiglitazone or GW9662 alone (Fig. 9).

## Discussion

The International Agency for Research on Cancer estimated that there were 9.6 million cancer deaths in 2018^52^. Such statistic indicates that cancer is the second most common cause of death worldwide after cardiovascular diseases. Therefore, there is an urgent need to find an effective cure for cancer. As 5-ene-2-amino(imino)-4-thiazolidinones showed a positive anticancer activity in a previous screening^16^, we decided to study the anticancer potential of 5Z-(4-fluorobenzylidene)-2-(4-hydroxyphenylamino)-thiazol-4-one (Les-236) in the following four human cancer cell lines: squamous cell carcinoma (SCC-15), lung carcinoma (A549), colon adenocarcinoma (CACO-2), and neuroblastoma (SH-SY5Y). Our experiments showed a decrease in ROS production in all the studied cancer cell lines (SCC-15, CACO-2, A549, and SH-SY5Y) and the normal skin fibroblast cell line (BJ) under the action of Les-236. The strongest decrease in ROS production in all the time intervals was observed in CACO-2 cell line, while SH-SY5Y cell lines showed the least sensitivity to Les-236. Moreover, in all studied cell lines, cell co-treatment with H_2_O_2_ and Les-236 showed that Les-236 reduced ROS production stimulated by H_2_O_2_. Currently, there is evidence that the mechanism of anticancer activity of structurally similar 4-thiazolidinones is associated with increase in the level of ROS^19,37^. However, our data suggest a different mechanism for the activity of Les-236. Similar results have been previously obtained by Коbylinska *et al*. (2016) who studied the activity of 5-heterylidene 2-(4,5-dihydropyrazol-1-yl)-4-oxo-4,5-dihydro-1,3-thiazoles in rat glioma (C6) cell line^53^. The compound studied by Коbylinska *et al*. (2017) also decreased ROS production in human glioma (U251) cell line^54^. Moreover, decrease in ROS production correlated with decrease in the activity of superoxide dismutase and increase in the activity of catalase^53^. Due to similiarities in chemical structure between the two compounds, we belived that Les-236 might act in a similar way.

Our study showed that despite decrease in production of ROS, the Les-236 compound exhibited cytotoxicity. The strongest induction of LDH release was observed in BJ, SCC-15, and SH-SY5Y cell lines. The resazurin reduction assay revealed that Les-236 decreased metabolism/proliferation in all the studied cell lines at micromolar concentrations, with the strongest reduction observed in SH-SY5Y cell line. All the tests showed that neuroblastoma was very sensitive to Les-236, as the compound caused necrosis in the SH-SY5Y cell line.

To date, it has been described that high *in vitro* concentrations (µM) of thiazolidinediones (TZDs), such as ciglitazone, rosiglitazone, pioglitazone, and troglitazone, lead to cell cycle arrest, apoptosis, and redifferentiation^42–44,55^, suggesting a putative link between PPARγ signaling and the antitumor activities of TZDs. The mentioned TZDs were shown to increase LDH release in mouse glioma cells (GL261), C6, human colorectal adenocarcinoma (HT29), and human colon carcinoma (COLO-205) cell lines^56,57^, as well as decrease cell metabolism/proliferation in human breast cancer (MCF-7), A549, and human prostate cancer (PC-3) cell lines^19,58,59^. Furthermore, the *in vivo* anticancer efficacy of troglitazone was demonstrated in a few clinical studies that involved patients with liposarcomas or prostate cancer^45^. Similar to our study, Коbylinska *et al*.^53,54^ showed that a new derivate of 4-thiazolidinone (Les-3288) decreased cell metabolism/proliferation as revealed by the MTT assay in C6 and U251 cells^53,54^. Our previous studies showed that 4-thiazolidinone derivatives (Les-2194, Les-3377, Les-3640) caused LDH release and decrease of resazurin reduction in SCC-15 cell lines^37^. Moreover, PPARγ silencing reduced the toxicity of related thiazolidinones (Les-2194, Les-3377, Les-3640) which confirms the crucial role of PPARγ receptors in the mechanism of action of 4-thiazolidinone derivatives^20^.

The most important marker of apoptosis is activation of caspase-3 which results in DNA fragmentation, degradation of the cytoskeleton and nuclear proteins, cross-linking of proteins, and finally, formation of apoptotic bodies^60^. Our study showed that after 6 h of exposure to Les-236, caspase-3 activity increased in the BJ cell line at all studied concentrations. In cancer cell lines (SCC-15, CACO-2, A549, and SH-SY5Y), caspase-3 activity was increased only at micromolar concentrations after 6 and 24 h of exposure to Les-236. Interestingly, after 48 h of exposure, Les-236 caused caspase-3 activation at all studied concentrations in A549 (excluding 100 µM which is a highly necrotic concentration), CACO-2, and SCC-15 cell lines. The observation that Les-236 also caused caspase-3 activation in normal skin fibroblasts (BJ) is disturbing. The long duration of exposure needed for apoptosis activation in A549, CACO-2, and SCC-15 cell lines suggests the complex mechanism of action of Les-236 and its promising effect in cancer treatment. Moreover, in A549, CACO-2, and SCC-15 cell lines, apoptosis correlated with increase in expression and/or activation of PPARγ^20,61,62^. Our previous study showed that Les-3377 and Les-3640 increased caspase-3 activity in SCC-15 cells, and silencing of PPARγ prevented the activation of caspase-3^20,37^.

The half-maximal inhibitory concentration (IC_50_) is the most widely used and informative measure of a drug’s efficacy. It indicates how much drug is needed to inhibit a biological process by half, thus providing a measure of the potency of an antagonist drug in pharmacological research. As measured by resazurine reduction, Les-236 has the highest potential to inhibit cell metabolism/proliferation in the SH-SY5Y cell line (0.24 µM) and the weakest in the A549 cell line (2062.00 µM) (Table 1). The half maximal effective concentration (EC_50_) level is a well-known quantity used in pharmacology and toxicology to calculate the efficacy of a substance from a dose–response curve. It represents the dose at which 50% of the maximum efficacy is observed. EC_50_ values were calculated for LDH release and caspase-3 activity to determine the dose-dependent effect on lethality to cells. The lowest EC_50_ for LDH release was for the SH-SY5Y (0.37 µM) cell line. For caspase-3 assay, the lowest EC_50_ was for the CACO-2 cell line (10.36 µM).

In the second part of our study we showed that after cell exposure to 1 µM Les-236, KI67 protein (considered as a marker of proliferation) expression significantly decreased in BJ and SH-SY5Y cell lines which correlates with decreased resazurine reduction. In the mentioned cell lines, co-stimulation with Les-236 and rosiglitazone (PPARγ agonist) or GW9662 (PPARγ antagonist) did not affect expression of KI67 decreased by Les-236. In CACO-2, SCC-15 and A549 cell lines, 1 µM Les-236 did not affect KI67 protein expression. In the CACO-2 cell line, cell co-stimulation with Les-236 and rosiglitazone increased expression of KI67. In the A549 cell line, co-treatment with Les-236 and GW9662 decreased KI67 protein expression. Interestingly, in the SCC-15 cell line, co-stimulation with both, Les-236 and rosiglitazone and Les-236 and GW9662, increased KI67 protein expression. As mentioned above, in *in vitro* studies, high concentrations (µM) of TZDs, led to cell cycle arrest, apoptosis, and redifferentiation^42–44,55^. However, in some cell lines, such as SCC, A549 and CACO-2, TZDs can stimulate cells proliferation depending on the concentration^63,64^. Our experiments show that after a 6 h exposure, Les-236 affects *PPARγ* mRNA expression in all studied cell lines. Moreover, Les-236 alone or in co-treatment with rosiglitazone or GW9662, affects expression of PPARγ protein in the studied cell lines. In CACO-2, A549, and SH-SY5Y cell lines, Les-236 acts similar to GW9662. In the SCC-15 cell line, Les-236 acts similar to rosiglitazone. Unfortunately, in the BJ cell line, results are not conclusive and we cannot clearly determine whether Les-236 works similar to an agonist or an antagonist of PPARγ. We suggest that in the A549, CACO-2, and SCC-15 cell lines, PPARγ-dependent pathways could be involved in cell death caused by Les-236; however, more studies are needed to confirm this hypothesis.

In conclusion, our results showed the anticancer potential of 5*Z*-(4-fluorobenzylidene)-2-(4-hydroxyphenylamino)-thiazol-4-one (Les-236) toward the human cancer cell lines. The studied compound decreased ROS production in BJ, SCC-15, CACO-2, A549, and SH-SY5Y cell lines at micromolar concentrations. However, the compound increased LDH release and decreased cell metabolism/proliferation at micromolar concentrations in BJ, SCC-15, CACO-2, A549, and SH-SY5Y cell lines. Les-236 was able to increase the activity of caspase-3 in BJ (after 6 h of exposure), A549, CACO-2, and SCC-15 (after 48 h of exposure) cell lines over a wide range of concentrations (1 nM to 100 µM), which could be due to the activation of PPARγ-dependent pathways. However, the safety of this compound and its metabolites should be examined in other normal human cells. Therefore, further studies on the mechanism underlying the effects of Les-236 *in vitro* and *in vivo* are needed.

## Materials and Methods

### Reagents

Trypsin, penicillin, streptomycin, phosphate-buffered saline without Ca^2+^ and Mg^2+^, hydrocortisone, sodium pyruvate, sodium bicarbonate, dimethyl sulfoxide (DMSO), caspase-3 substrate (235400), 2′,7′-dichlorodihydrofluorescein diacetate (H_2_DCFDA) (D6883), GW9662, rosiglitazone, hydrogen peroxide (H_2_O_2_) and resazurin (R7017) were purchased from Sigma-Aldrich (St. Louis, MO, USA). Black 96-well culture plates (655090) were purchased from Greiner Bio-One (Frickenhausen, Germany). Charcoal/dextran-treated fetal bovine serum (FBS), heat-inactivated FBS, RNA isolation kit (E0309), and fast probe real-time PCR master mix (Rox) (E0423) were purchased from EURx (Gdańsk, Poland). F12K, MEM, and DMEM/F12 (1:1) media were purchased from Corning (Manassas, VA, USA). The lactate dehydrogenase (LDH) cytotoxicity detection kit (11644793001) was purchased from ROCHE Applied Science (Mannheim, Germany). The High Capacity cDNA Reverse Transcription Kit and the TaqMan® probes corresponding to specific genes encoding *β-Actin* (Hs01060665_g1) and *PPARγ* (Hs00234592_m1) were purchased from Life Technologies (Forest City, CA, USA). PPARγ (H1361) and KI67P (H5432) ELISA assays were obtained from Elabscience Biotechnology (WuHan, China).

The synthesis and physicochemical properties of the tested compound 5*Z*-(4-fluorobenzylidene)-2-(4-hydroxyphenylamino)-thiazol-4-one (Les-236) were described previously^16^ (Fig. 1). This compound was selected based on the results of a previous screening^16^.

### Cell culture

Human squamous cell carcinoma (SCC-15), lung carcinoma (A549), colon adenocarcinoma (CACO-2), neuroblastoma (SH-SY5Y), and skin fibroblast (BJ) cell lines were obtained from the American Type Culture Collection (ATCC, distributor: LGC Standards, Łomianki, Poland). The A549 cell line was maintained in the F12K medium, SCC-15 and SH-SY5Y cell lines were maintained in the DMEM/F12 medium and BJ and CACO-2 cell lines were maintained in the MEM medium. The media used in the experiments were phenol red-free and contained 2.5 mM L-glutamine. Moreover, the media were supplemented with 10% FBS (or heat-inactivated FBS for SH-SY5Y cells) and 400 ng/mL hydrocortisone (only for SCC-15 cells). The cells were maintained in an incubator at 37 °C in a humidified atmosphere with 5% CO_2_. For ROS measurements, lactate dehydrogenase (LDH) release, resazurine reduction and caspase-3 activity assays, the cells were seeded in 96-well culture plates at a density of 6 × 10^3^ (for 6 and 24 h treatments) and 5 × 10^3^ (for 48 h treatment) per well and initially cultured before the experiment for 24 h. For the real-time PCR assay, different cell lines were seeded onto 12-well plates and initially cultured for 24 h. For the protein measurement, different cell lines were seeded onto 6-well plates and initially cultured for 24 h. Subsequently, the medium was replaced with a fresh one by raising the concentrations (1, 10, 50, and 100 nM and 1, 10, 50, and 100 µM) of the studied compound Les-236.

### Estimation of ROS production

To determine the ability of Les-236 to induce ROS production in the cells, 5 μM H_2_DCFDA was applied. The cells were incubated with H_2_DCFDA in serum-free and phenol red-free medium for 45 min before treatment with increasing (1, 10, 50, and 100 nM and 1, 10, 50, and 100 µM) concentrations of the studied compound according to a previously described method^65^. After incubating the cells for 3, 6, and 24 h with increasing concentrations of the compound (and 5% CO_2_ at 37 °C), the culture medium was replaced with a fresh one to remove extracellular residual dichlorodihydrofluorescein (DCF). Cells treated with 100 µM hydrogen peroxide (H_2_O_2_) were used as a positive control (results not shown). DCF fluorescence was measured using a microplate reader (FilterMax F5) at the maximum excitation and emission spectra of 485 and 535 nm, respectively.

### LDH cytotoxicity assay

The cytotoxicity detection kit is used for colorimetric assay to quantify the cell death -necrosis and secondary necrosis (after the disintegration of the apoptotic bodies in the cell culture environment)^66^. The assay was based on cell lysis and the release of LDH from the cytosol of the damaged cells into the surrounding medium. After 6, 24, and 48 h of treatment of the cells with increasing concentrations of the studied compound, 100 µL of the culture supernatants was collected and incubated in the reaction mixture from the kit according to the manufacturer’s protocol. After 30 min, the reaction was stopped by adding 1 N HCl and the absorbance was measured at a wavelength of 490 nm using the FilterMax F5 Multi-Mode microplate reader (Molecular Devices, Corp., Sunnyvale, CA, USA).

### Resazurin reduction cell viability and metabolism assay

A working solution of 60 µM resazurin was prepared in a medium containing 1% FBS on the day of analysis according to a previously described method^37^. After 6, 24, and 48 h of treatment of cells with increasing concentrations of the Les-236 compound in 96-well culture plates, the cells in the wells were replaced by a working solution of resazurin (100 µL) and the plates were incubated at 37 °C. Fluorescence was measured at an excitation wavelength of 530 nm and an emission wavelength of 590 nm on the FilterMax F5 Multi-Mode microplate reader (Molecular Devices, Corp., Sunnyvale, CA, USA) after 30 and 60 min of dye addition.

### Caspase-3 activity

Caspase-3 activity was used as a marker of cell apoptosis. After 6, 24, and 48 h of treatment of cells with increasing concentrations of Les-236, the culture plates were frozen at −80 °C and left until assay. The frozen cells were lysed with lysis buffer (50 mM HEPES (pH 7.4), 100 mM NaCl, 0.1% CHAPS, 1 mM EDTA, 10% glycerol, and 10 mM DTT) at 10 °C for 10 min according to a previously described protocol^37^. The lysates were incubated with caspase-3 substrate Ac-DEVD-pNA at 37 °C. Cells treated with 1 μM staurosporine were used as a positive control (results not shown). After 30 min, the absorbance of the lysates at 405 nm was measured using the FilterMax F5 Multi-Mode microplate reader. The amount of colorimetric product produced was continuously monitored for 120 min. The data were analyzed using Multi-Mode Analysis software (Molecular Devices, Corp., Sunnyvale, CA, USA) and were normalized to absorbance of the vehicle-treated cells.

### Real-time PCR analysis of mRNAs specific to genes encoding *PPARγ*

The experiment was conducted as per a previously described procedure^20^. After a 6 h exposure to 1 or 10 µM Les236, the samples were collected and total RNA was extracted from the cells using an RNA isolation kit according to the manufacturer’s instructions. The quality and quantity of the RNA were determined spectrophotometrically at 260 and 280 nm, respectively (NanoDrop ND/1000 UV/Vis, Thermo Fisher, USA). Two-step real-time reverse transcription (RT) PCR was conducted, with both the RT reaction and the quantitative PCR (qPCR) run using the CFX Real Time System (BioRad, USA). The RT reaction was carried out at a final volume of 20 μL with 250 ng RNA (as a cDNA template) using the cDNA reverse transcription kit in accordance with the manufacturer’s instructions. Products from the RT reaction were amplified using the fast probe qPCR master mix (Rox) kit with TaqMan probes as primers for specific genes encoding *ACTB* and *PPARγ*. Amplification was carried out in a total volume of 25 μL containing 1 × fast probe qPCR master mix (Rox) and 1.0 μL of the RT product which was used as the PCR template. Standard qPCR procedures were carried out as follows: 2 min at 50 °C and 10 min at 95 °C, followed by 45 cycles of 15 s at 95 °C and 1 min at 60 °C. The threshold value (Ct) for each sample was set during the exponential phase, and the ΔΔCt method was used for data analysis. *ACTB* was used as the reference gene.

### ELISA for PPARγ and KI67

The levels of PPARγ and KI67 proteins were determined through ELISA after a 48 h of treatment with 1 µM Les236 or co-treatment with Les-236 and 10 µM rosiglitazone or Les-236 and GW9662. These proteins were specifically detected with ELISA and subsequently subjected to quantitative sandwich enzyme immunoassay, which was conducted according to the manufacturer’s instructions (Elabscience Biotechnology, Wuhan, China). Briefly, a 96-well plate was pre-coated with monoclonal antibodies specific to PPARγ and KI67. Standards and the collected cell extracts were added to the wells and incubated for 90 min at 37 °C. Next, the liquid was removed, and biotinylated detection antibodies (100 µL) were added to the cultures for 60 min. We washed the cells three times to remove any unbound substances, and then added horseradish peroxidase-conjugated avidin. The cells were washed again, and 90 µL of substrate solution was added to the wells for 15 min. Then, 50 µL stop solution was added to terminate the reaction and the absorbance was measured at 450 nm; the value obtained was proportional to the amount of PPARγ and KI67, respectively. The total protein concentration was determined in triplicate in each sample by using a Thermo Fisher NanoDrop device.

### Statistical analysis

The data are presented as means ± SD of three independent experiments. Each treatment was repeated six times (n = 6), and the values were measured in triplicate. The average of the triplicate samples was used for the statistical analyses. Considering the difference in data obtained from the measurement of fluorescence and absorbance, the results were presented as a percentage of the controls. The data were analyzed using one-way analysis of variance followed by Tukey’s multiple comparison procedure; ^*^p < 0.05, ^**^p < 0.01, and ^***^p < 0.001 vs. control cells were considered significant. Statistical significance is marked on the figures (Figs 3, 7, 8, 9) or in the supplementary data (Figs 2, 4, 5, 6).

### Highlights

- Les-236 decreased the production of reactive oxygen species in BJ, SCC-15, CACO-2, A549, and SH-SY5Y cell lines.
- At high micromolar concentrations, Les-236 increased the release of lactate dehydrogenase in all the studied cell lines.
- Les-236 activated caspase-3 in A549, CACO-2, and SCC-15 cell lines at all the studied concentrations.
- PPARγ is involved in Les-236 mechanism of action.

## Supplementary information

Supplementary data
